# Decoding auditory spatial and emotional information encoding using multivariate versus univariate techniques

**DOI:** 10.1007/s00221-018-5185-7

**Published:** 2018-01-27

**Authors:** James H. Kryklywy, Ewan A. Macpherson, Derek G. V. Mitchell

**Affiliations:** 1 Department of Psychology, University of British Columbia, Vancouver, V6T 1Z4 Canada; 2 Graduate Program in Neuroscience, University of Western Ontario, London, ON N6A 5A5 Canada; 3 Brain and Mind Institute, University of Western Ontario, London, ON N6A 5B7 Canada; 4 School of Communication Sciences and Disorders, University of Western Ontario, London, ON N6G 1H1 Canada; 5 National Centre for Audiology, University of Western Ontario, London, ON N6G 1H1 Canada; 6 Department of Anatomy and Cell Biology, University of Western Ontario, London, ON N6A 3K7 Canada; 7 Department of Psychiatry, University of Western Ontario, London, ON N6A 5A5 Canada

**Keywords:** Emotion, Localization, Auditory pathways, fMRI, MVPA searchlight

## Abstract

Emotion can have diverse effects on behaviour and perception, modulating function in some circumstances, and sometimes having little effect. Recently, it was identified that part of the heterogeneity of emotional effects could be due to a dissociable representation of emotion in dual pathway models of sensory processing. Our previous fMRI experiment using traditional univariate analyses showed that emotion modulated processing in the auditory ‘what’ but not ‘where’ processing pathway. The current study aims to further investigate this dissociation using a more recently emerging multi-voxel pattern analysis searchlight approach. While undergoing fMRI, participants localized sounds of varying emotional content. A searchlight multi-voxel pattern analysis was conducted to identify activity patterns predictive of sound location and/or emotion. Relative to the prior univariate analysis, MVPA indicated larger overlapping spatial and emotional representations of sound within early secondary regions associated with auditory localization. However, consistent with the univariate analysis, these two dimensions were increasingly segregated in late secondary and tertiary regions of the auditory processing streams. These results, while complimentary to our original univariate analyses, highlight the utility of multiple analytic approaches for neuroimaging, particularly for neural processes with known representations dependent on population coding.

## Introduction

In vision, emotional information augments activity within sensory cortices (Vuilleumier and Driver 2007) and alters stimulus detection (Graves et al. 1981) and awareness (Amting et al. 2010; Mitchell and Greening 2011). Similarly, the auditory system confers preferential access to neural resources for emotional auditory stimuli (Fecteau et al. 2007; Ethofer et al. 2012). Although there have been numerous studies that seek to identify the ‘what’ of auditory emotion (i.e., identifying auditory properties that carry emotional information; Lieberman and Michaels 1962; Protopapas and Lieberman 1997), very little work has investigated the role of emotion on naturalistic auditory spatial feature processing. Auditory processing occurs within two separate cortical streams (Rauschecker and Tian 2000; Alain et al. 2001; Lomber and Malhotra 2008) diverging from Heschl’s gyrus (HG; Ahveninen et al. 2006) adjacent to primary auditory cortex (A1; Rauschecker and Romanski 2011). An anterior lateral ‘what’ pathway for identity processing has been identified in anterior STG and inferior frontal lobe. In contrast, a posterior medial ‘where’ pathway implicated in processing auditory spatial cues for sound localization includes posterior superior temporal gyrus (STG), inferior parietal lobe, and superior frontal sulcus. Recently, it has been suggested that emotion may have a differential effect across these streams (Kryklywy et al. 2013), influencing neural activity in the auditory ‘what’ stream, but not the ‘where’ stream during sound localization, with minimal areas of overlap between these representations. Traditional univariate fMRI analysis, however, may not be optimal for delineating auditory stimulus representation, and may have under-estimated the impact of emotion on spatial processes. This is important given the conflicting reports in the literature concerning the extent to which dorsal stream processes are modulated by emotion (Goldberg et al. 2014).

Since our prior publication (Kryklywy et al. 2013), multivariate analyses, such as multi-voxel pattern analyses (MVPA), have been established as an important tool for determining how neural patterns code mental representations, and appear to compliment univariate approaches with increased sensitivity (Norman et al. 2006; Mahmoudi et al. 2012). They are particularly beneficial for delineating population-based neural encoding, like that found in auditory processing (Mizrahi et al. 2014). Furthermore, multivariate analyses appear to be less sensitive to between-subject variance than traditional univariate analyses (Davis et al. 2014). Therefore, it may be that the observed areas exhibit a higher level of between-subject variance in gross changes in net activation, resulting in the effects being missed by the univariate technique, but still detected by MVPA.

The current work aims to re-examine the impact of emotion on the ‘what’ and ‘where’ streams of auditory processing using MVPA applied to our prior data (Kryklywy et al. 2013). A spherical searchlight approach was selected to identify patterns of activity representation for auditory emotional- and location-related information during an auditory localization task. Results were compared with those presented in our previous univariate analyses. With the additional sensitivity to representation coding afforded by multivariate analyses techniques used in the current study, we expected to see greater representation of both emotion and spatial features in early auditory cortices when contrasted with prior univariate analyses.

## Methods

### Subjects

18 healthy human subjects, (9 male, 9 female) with a mean age of 23.56 (range 19–35, SD 4.51), completed the emotional localization task. All subjects granted informed consent and were in good mental health, as assessed by a Structured Clinical Interview for DSM-IV (*Diagnostic and Statistical Manual of Mental Disorders*, 4th Edition). All subjects reported normal hearing, normal or corrected-to-normal vision, no known neurological disorders, and were fluent English speakers. All experiments were approved by the Health Science Research Ethics Board at the University of Western Ontario.

### Stimuli and apparatus

#### Stimuli

Twelve sounds were chosen from the *International* *Affective Digitized Sound* (IADS) stimulus set that were of a neutral, negative or positive affective nature as defined by standardized ratings (Bradley and Lang 1999). To ensure generalizability of the results and reduce the potential impact of habituation, multiple stimuli were used for each emotional category that were statistically balanced across multiple emotional and auditory attributes. Positive and negative stimuli were balanced for arousal ratings (mean positive = 6.58, mean negative = 6.92) and valence levels (positive = 7.56, negative = 2.36, absolute neutral = 5). Each stimulus category contained two single-source non-verbal human vocalizations, one multi-source non-verbal human vocalization, and one non-human sound. Importantly, stimuli were normalized such that each stimulus had the same root-mean-square level computed on the concatenated left- and right-ear signals, which ensures that the power and energy were consistent. To facilitate deconvolution of the BOLD signal, three versions of each sound were produced corresponding to three unique durations: 2000, 2500 and 3000 ms. Volume was controlled by a Dayton Audio Class T Digital Amplifier. Initial volume was set to ~ 89 dBs and adjusted slightly to the comfort level of each individual participant.

#### Auditory virtual environment

All sounds were presented within an auditory virtual environment through Sensimetric MRI-Compatible Insert Earphones. To induce the perceptual experience of spatialized sounds using insert-style headphones, HRTFs were measured individually for each subject. A detailed procedure of the creation of auditory virtual environments can be found in Kryklywy et al. (2013).

Sounds for the emotion localization task were spatialized to four locations along the horizontal plane (− 90°, − 22.5°, 22.5° and 90° from the sagittal midline; negative = left). The entire set of stimuli (across locations, sound sources, and listeners) was normalized such that each stimulus had the same root-mean-square level computed on the concatenated left- and right-ear signals.

### Procedure

**Fig. 1:**
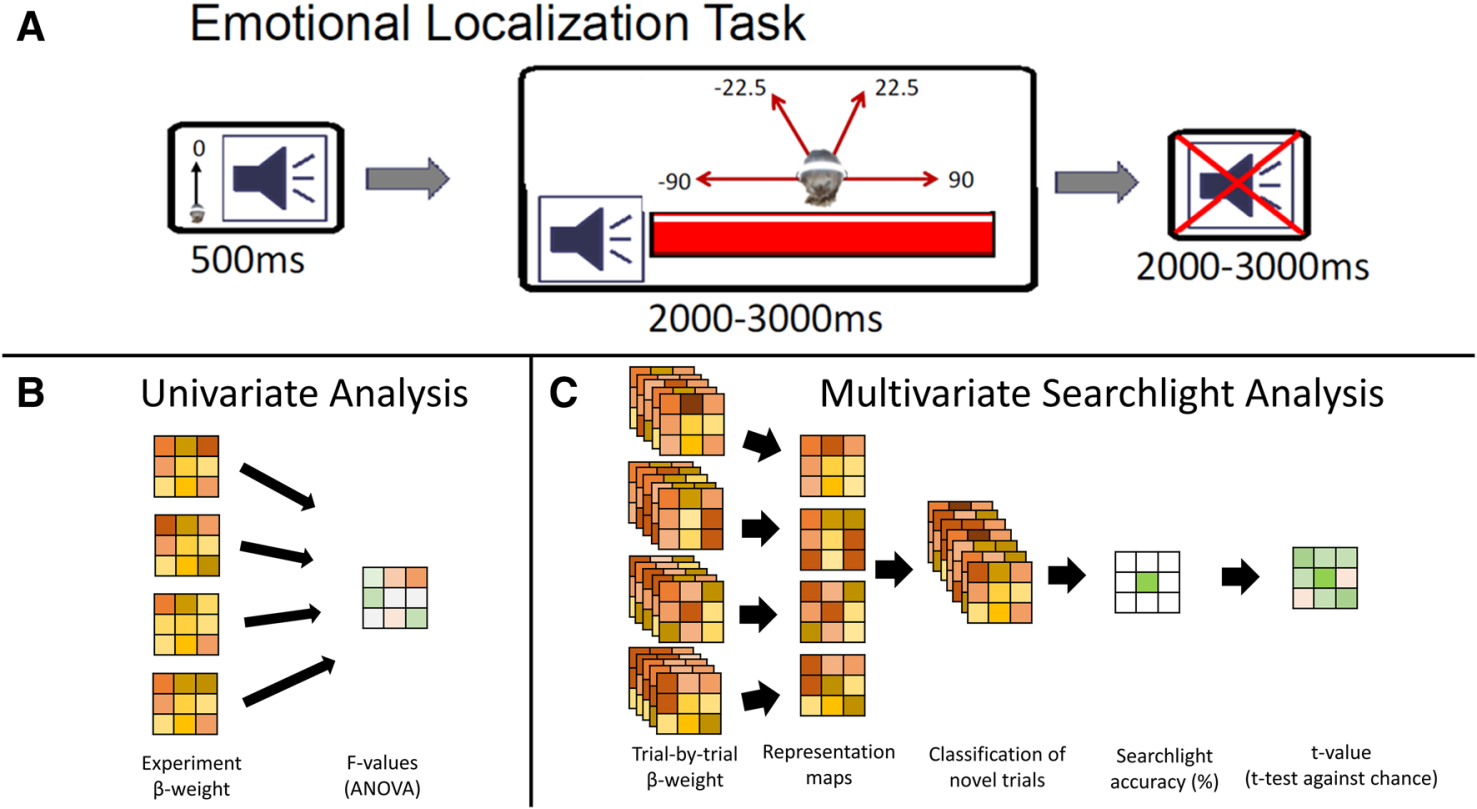
Auditory localization of emotional cues. **a** For each trial of the auditory emotional localization task, participants were presented with an auditory fixation cross followed by a target stimulus. This stimulus was presented from one of four virtual locations, and consisted of either a neutral, negative or positive sound. During the sound presentation, a response was made to indicate the sound location via button press. Following each trial, there was a 2000–3000 ms period of silence (as depicted by the superimposed red X on the right). **b** Univariate statistics were performed on whole brain EPI data comprised of β-weights for each voxel derived from modeling the entire experimental time course. **c** N-1 classification was performed in a 10 mm spherical searchlight on β-weights derived from modeling each stimulus presentation independently. Classification accuracy for each searchlight was assigned to its centre voxel. Resultant accuracy maps were contrasted against chance performance for classification

Procedural details for the emotional auditory localization task are presented in Fig. 1a. In brief, each trial consisted of a white noise burst spatialized to the participant’s sagittal midline (acting as a central ‘auditory fixation cross’), a spatialized target sound presented in one of four locations randomly, and a period of silence. Participants located the target sound via button press. Each sound was presented from each location six times (288 valid trials; 96 per emotion condition), and 96 silent ‘jitter’ trials, for a total of 384 trials broken over six equal runs. Participants closed their eyes for the duration of each scan to reduce possible confounds of visual feedback.

### Imaging

#### MRI data acquisition

Subjects were scanned during all task performances using a 3T Siemens Scanner with a 32-channel head coil. fMRI images were taken with a T2*-gradient echo-planar imaging sequence [repetition time (TR) = 2500 ms, echo time (TE) = 36 ms; field of view (FOV) = 18.8 cm, 78 × 78 matrix]. For all functional runs, complete brain coverage was obtained with 38 interleaved slices of 2.4 × 2.4 mm in plane with slice thickness of 2.4 mm, forming 2.4 mm isovoxels (148 functional images for each run). A high-resolution T1-weighted anatomical scan was obtained covering the whole brain (TR = 2300 ms, TE = 4.25 ms; FOV = 25.6 cm, 192 axial slices; voxel size = 1 mm isovoxels; 256 × 256 matrix).

#### Preprocessing and multivariate fMRI analysis

Initial univariate analysis of the fMRI data (Fig. 1b) was conducted using Analysis of Functional NeuroImages (AFNI) software (Cox 1996) at both the individual and group levels, and has been described elsewhere (Kryklywy et al. 2013). Following all initial analyses, multivoxel pattern analysis (MVPA) utilizing whole-brain spherical searchlights (Fig. 1c) was performed on the data from the emotional auditory localization task consisting of regressor files modeling the time course of relevant stimuli for each of the 12 conditions during correct emotional localization trials (4 locations × 3 emotions).

All MVPA analyses were implemented in the Princeton MVPA Toolbox for Matlab. Analyses were conducted using a data-driven searchlight MVPA approach using 10 mm searchlight radii. 3dDeconvolve was used to determine a β coefficient for each voxel on each individual trial over all six functional runs of the experimental task. In addition, the AFNI function 3dSkullStrip was used to generate a mask from each participant’s anatomical scan containing only grey matter voxels in native space. To ensure that no grey matter voxels were inadvertently removed, this mask was dilated two levels using the AFNI function 3dmask_tool. All functional data used in subsequent MVPA analyses were filtered by these anatomical masks.

Classification procedures were performed twice for each searchlight; first using functional data modeling the location of the auditory stimuli, and second using EPI data modeling the emotional content. The time series data were normalized by dividing the signal intensity of a voxel at each time point by the mean signal intensity of that voxel for each run and multiplying the result by 100. Thus, resultant regression coefficients represent the percent signal change from the mean activity. Two sets of regressor files were generated to model the presentation time course of emotion-related and location-related activity individually for each sound presentation. The relevant hemodynamic response function was fit to each regressor to perform linear regression modeling. To account for voxel-wise correlated drifting, a baseline plus linear drift and quadratic trend were modeled for each time series. This resulted in two β coefficients (emotion- and location-dependent) for each voxel and regressor. All data used in this level of analysis were unsmoothed and in native space. To correct for multiple comparisons, a spatial clustering operation was performed using 3dClustSim (v2015) with 1000 Monte Carlo iterations on the whole brain EPI matrix. In light of recent concerns about inflated false positives in fMRI analysis (see Eklund et al. 2016), a stringent cluster-defining threshold of *p* < 0.001 was used, and corrected to *p* < 0.05.

Spherical searchlights were generated centred on each voxel containing β coefficients for all voxels in a 10 mm radius from this centre point. To minimize potential conflicts, searchlight specifications, including radius, were chosen a priori, in consultation with previous searchlight procedures performed in auditory cortex (Linke et al. 2011; Du et al. 2014). Classification was performed within each searchlight using an n-1 back-propagation classification procedure (i.e., training the classifier on 5 of 6 runs, before testing the remaining run for all potential combinations). The predictive accuracy for each searchlight was assigned to the centre voxel. This resulted in six separate maps of predictive accuracy for each participant, representing each permutation of the n-1 procedure. Outputs were averaged across runs using the AFNI function 3dcalc and transformed into the standard space of Talairach and Tournoux. Group analyses were then performed to determine where the predictive accuracy of our classifier was significantly greater than chance (i.e., > 33.3% for emotion data and > 25% for location data). Following this, a series of conjunction analyses were performed using 3dcalc (AFNI) to determine the amount of overlap between the emotion- and location-predictive regions in the current analyses, and to contrast these results with a previously published univariate analysis of the data (Kryklywy et al. 2013). Jaccard similarity indices (JI; 1912) were calculated for each conjunction to help quantify the degree of overlapping activation observed between conditions, and between the results of the univariate and multivariate analyses. JIs were computed as the shared voxel space in a conjunction divided by the total voxels activated by both activations of interest (i.e., JI = [volume A∩B]/[volume A∪B]).

## Results

### Behavioural and univariate imaging results

All behavioural results were published previously (Kryklywy et al. 2013). In summary, a significant main effect of emotion was identified in the reaction time data characterized by significantly slower localization of positive and negative sounds compared to neutral sounds (*p* < 0.001 and *p* < 0.05 respectively). No other significant effects of emotion or emotion by location interactions were identified in the reaction time or accuracy data.

Univariate analyses were conducted by modeling the presentation time course of relevant stimuli created for each of the 12 conditions of the experimental task (4 locations × 3 emotions) for correct trials only, thus extracting the relevant β coefficient and t value for each voxel and condition (full details found in Kryklywy et al. 2013). A whole-brain analysis by way of a 4 (location) × 3 (emotion) ANOVA was conducted on these data to identify neural regions where activity varied as a function of location and emotion. In summary, this revealed that enhanced activity was elicited by positive and negative stimuli (versus neutral stimuli) in anterior–lateral areas of auditory cortex irrespective of sound location. In contrast, posterior–medial regions of auditory cortex, as well as the inferior parietal lobule and precuneus, were modulated by location, irrespective of emotion. A significant interaction effect was identified in a small cluster along primary auditory cortex. For extended results, please refer to Kryklywy et al. (2013).

### Imaging results: MVPA

#### MVPA: searchlight results

**Fig. 2:**
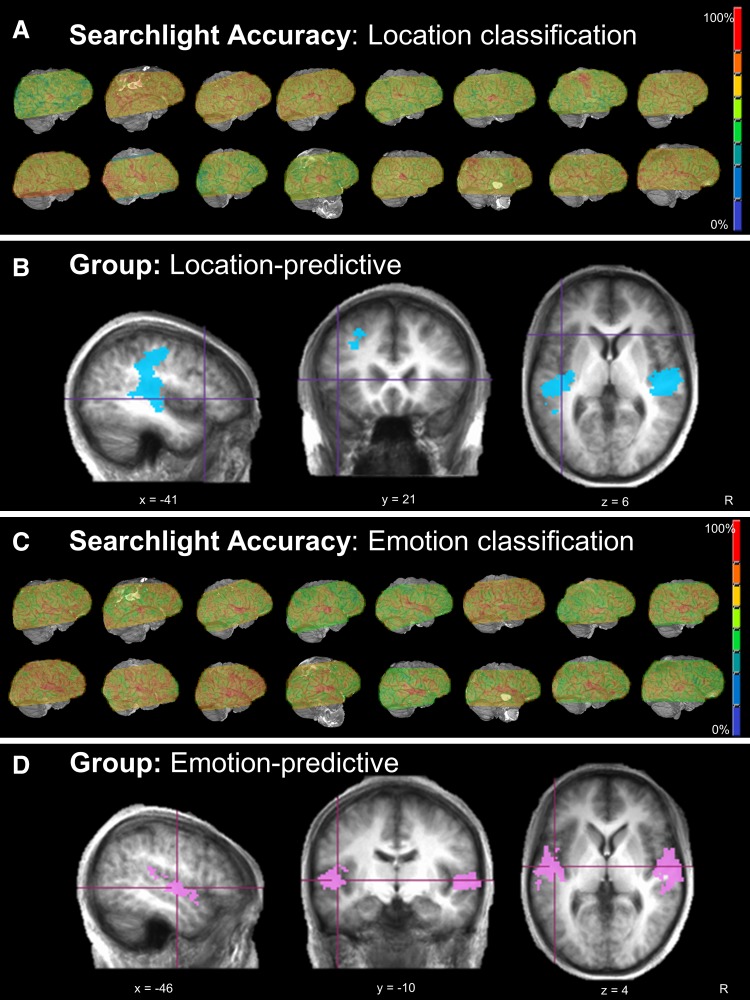
Neural regions displaying activity patterns predictive of location and emotion: 10 mm searchlight MVPA. **a** Classification accuracy for location information during the searchlight MVPA shown for each participant in native space. Accuracy for each voxel represents a 10 mm searchlight centred on that voxel, and is the average accuracy for all iterations of the *n* − 1 classifier procedure. **b** Group analysis demonstrates that sound location is predicted by patterns of activation within large regions of bilateral STG extending into the pre- and post-central gyrus. In addition, activity in regions of middle frontal and cingulate cortex were also predictive of sound location. **c** Classification accuracy for emotional information during the searchlight MVPA shown for each participant in native space. **d** The emotional nature of a sound was predicted by patterns of activity in bilateral STG at the group level

MVPA using 10 mm spherical searchlights identified patterns of activity predictive of location and emotion (Table 1; Fig. 2). Particularly, voxel activity patterns in bilateral STG predicted both the location and emotion of the sound at a level significantly above chance, with anterior regions of this area predictive only of the emotional information. In addition, middle frontal gyrus, posterior cingulate cortex and extensive regions of bilateral post- and pre-central gyrus were predictive of sound location.

**Table 1:** MVPA searchlight results

Predictive	R/L	Location	BA	*X*	*Y*	*Z*	Vol. (mm^3^)
Location	L	STS/TTG/PreCG/PostCG	2/4/13/41/42	−46	−25	20	18,784
	R	STS/TTG/PreCG/PostCG	2/4/13/41/42	48	−22	15	16,312
	L	MFG	8	−28	17	36	1288
	L	pCC	31	−14	−61	20	832
	R	CG	24	6	−14	43	568
	R	MOG	19	25	−84	22	376
	L	PhG	35	−31	−20	−15	248
Emotion	R	STG/Ins	13/22/41/42	54	−14	6	9264
	L	STG/Ins	13/22/41/42	−50	−14	6	7464

*STG* superior temporal gyrus, *TTG* transverse temporal tyrus, *MTG* middle temporal gyrus, *PostCG* post-central gyrus, *PreCG* pre-central gyrus, *MFG* middle frontal gyrus, *PCC* posterior cingulate cortex, *CG* cingulated gyrus, *MOG* middle occipital gyrus, *PhG* parahippocampal gyrus

*p* < 0.001, corrected to *p* < 0.05

#### MVPA and univariate: conjunction analysis

**Fig. 3:**
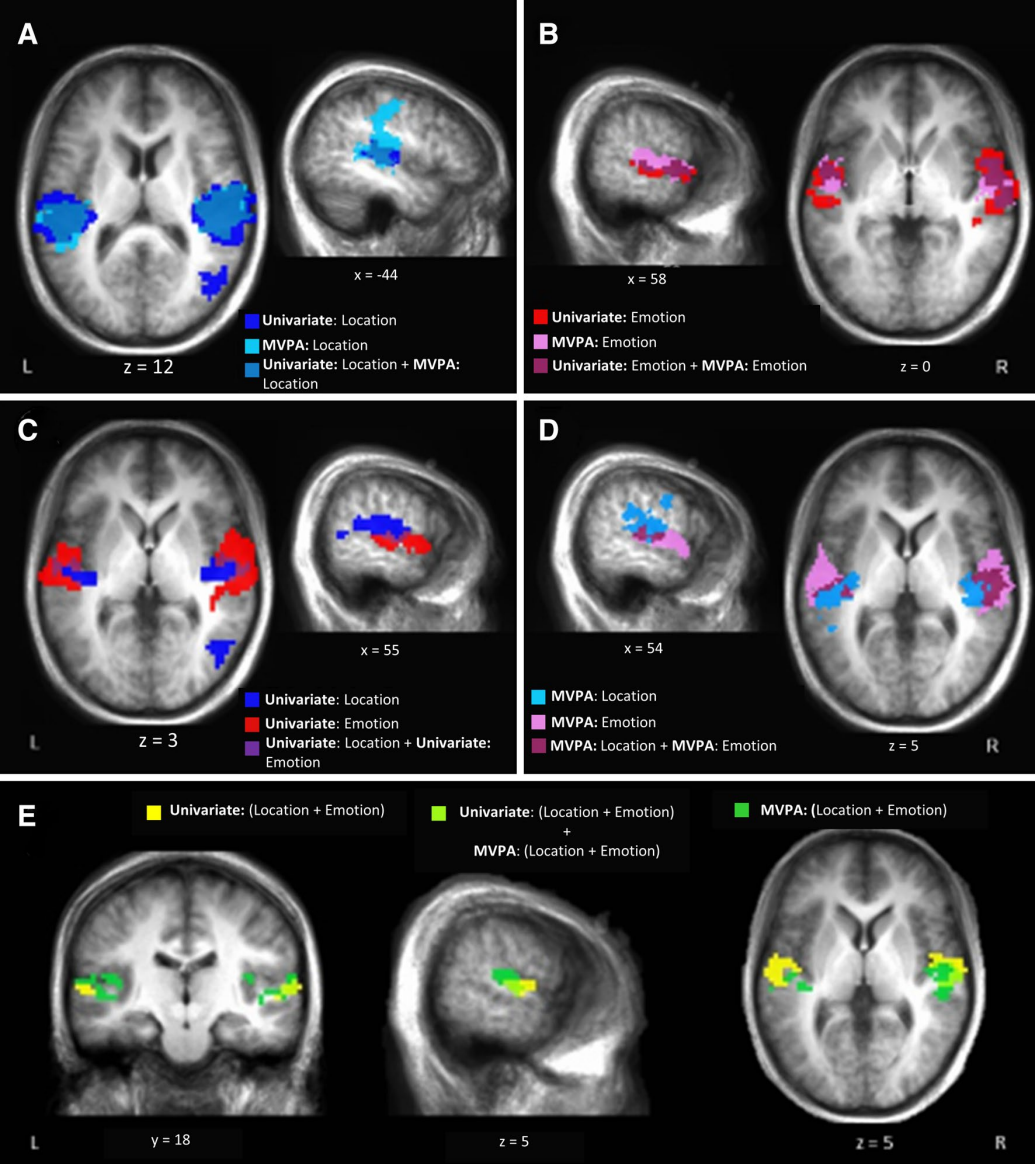
Distinct areas of superior temporal gyrus are responsive to emotion and location. **a, b** Conjunction of univariate and MVPA results for both sound location (blues) and emotion (red/pink) in the task. MVPA results were found to overlap with the analogous univariate results within secondary auditory areas, while also extending independently into both primary and tertiary auditory processing regions. **c, d** Independent conjunctions of univariate analysis and MVPA results. While distinct neural regions were identified as sensitive to individual auditory features in both analyses, the most extensive overlap was identified in the MVPA conjunction analysis. This overlap lay predominantly over regions involved in the early stages of auditory processing (BA 13/41). **e** Comparison of areas that were identified as being modulated by both location and emotion by each analytic technique. All univariate results taken from Kryklywy et al. (2013)

To help visualize the degree of overlap between univariate analyses conducted previously (Kryklywy et al. 2013) and the current MVPA analyses, a series of conjunction analyses were performed (Table 2). Along bilateral STG, large areas of overlap between regions identified as location sensitive by univariate analyses, and location predictive by MVPA, were identified (Fig. 3a). In addition, univariate emotion-sensitive regions were highly overlapping with regions identified by MVPA as emotion predictive (Fig. 3b).

**Table 2:** Conjunction of multivariate and univariate results

Conjunction	R/L	Location	BA	*X*	*Y*	*Z*	Vol. (mm^3^)	JI
Univariate: Location^a^	R	STG/TTG	13/41/42	48	− 23	13	11,178	0.28
+ MVPA: Location	L	STG/TTG	13/41/42	− 47	− 27	11	5913	
Univariate: Emotion^a^	R	STG	22	56	− 9	3	4752	0.40
+ MVPA: Emotion	L	STG	22	− 53	− 9	3	2754	0.33
Univariate: Emotion^a^	R	STG	22/41	56	− 12	6	1890	0.05
+ Univariate: Location^a^	L	STG	22/41	− 54	− 14	5	1512	0.11
MVPA: Location	R	STG/TTG/Ins	13/22/41/42	52	− 21	10	3744	0.16
+ MVPA: Emotion	L	STG/TTG/Ins	13/22/41/42	− 45	− 35	11	1960	0.07
Univariate: L∩E^a^	R	STG	41	51	− 21	9	567	0.12
+ MVPA: L∩E								

*STG* superior temporal gyrus, *TTG* transverse temporal gyrus, *Ins* Insula, *JI* Jaccard Index calculated for each hemisphere; [A∩B]/[A∪B]

^a^Data from Kryklywy et al. (2013) and Fecteau et al. (2007)

While minimal overlap was identified between areas showing univariate main effects of location and emotion (3402 mm3, JI = 0.06; Fig. 3c; Kryklywy et al. 2013), a greater overlap of representation was indicated by MVPA in both primary and early secondary auditory processing regions (5704 mm^3^, JI = 0.12; Fig. 3d). The relative overlap of emotion and location between univariate and MVPA were further examined, revealing limited similarity between these overlapping regions, even though they are both located within early auditory processing regions (567 mm^3^, JI = 0.07; Fig. 3e).

#### MVPA and univariate: region of interest (ROI) analysis

Due to the nature and limitations of MVPA searchlight analyses (Etzel et al. 2013), we performed an additional ROI-based MVPA classifier analysis. This aimed to determine whether the novel regions identified by the searchlight as containing both emotion- and location-related information did indeed distinguish between both conditions. As this region was already demonstrated to be location sensitive in both the original univariate and the current multivariate classifier, follow-up ROI analyses focused on its representation of emotional information.

ROI classification was performed using a similar procedure to that conducted in each individual searchlight (i.e., an *n* − 1 back-propagation classification procedure training the classifier on 5 of 6 runs, before testing the remaining run for all potential combinations). Prior to classification, however, all modelled EPI data was transformed in standardized space (Talairach and Tournoux) using the AFNI command adwarp. The predictive accuracy was then determined for two separate ROIs, one for each newly identified region of representational overlap in each hemisphere (Table 2). Within both ROIs, classifier performance was found to be significantly above chance (right: *t*_(15)_ = 11.750, *p* > 0.001, left: *t*_(15)_ = 9.939, *p* > 0.001). In addition, at a single-subject level, classifiers for all subjects were also found to perform significantly better than chance (all *p* < 0.005).

A second follow-up analysis was performed within these ROIs focusing on the percent signal change in the BOLD signal in response to the emotional categories. This supplementary analysis aimed to confirm that this area of representational overlap was indeed a novel result for MVPA, and that it could not be identified by univariate analyses alone. A one-factor repeated measures 1 × 3 (emotion: negative, neutral, positive) ANOVA was conducted on the percent signal change from each of the right and left ROI, as modelled by the original GLM (Kryklywy et al. 2013). No significant main effect of emotion was identified in either ROIs (right: *F*_(2, 18)_ = 1.173, *p* > 0.10, left: *F*_(2, 18)_ = 0.547, *p* > 0.10). Individually, while these analyses both have limitations (Kriegeskorte et al. 2009; Woo et al. 2014), together they support the notion of enhanced emotional representation in early location-sensitive auditory regions, and the utility of considering multivariate analytic techniques in conjunction with traditional univariate approaches.

## Discussion

Here, we extend prior work examining the impact of emotion on processing spatial characteristics of auditory stimuli (Kryklywy et al. 2013) by taking a multivariate approach featuring greater sensitivity to neural pattern representations and population coding. This identified regions of cortex predictive of both location and the emotional nature of auditory cues. Activity in posterior superior temporal gyrus, pre- and post-central gyrus and middle frontal gyrus was found to be predictive of sound location, while activity in anterior superior temporal gyrus, inferior frontal gyrus, insula and thalamus was found to be predictive of auditory emotion. These regions are consistent with the theorized correlates of the auditory ‘where’ and ‘what’ processing stream.

The current MVPA approach suggested that predictive coding of emotion and location in auditory processing streams are not as segregated in early processing areas as univariate analyses suggested (Kryklywy et al. 2013). This gain in representational overlap between emotion and location was identified primarily in early auditory regions associated with the putative ‘where’ auditory stream. In previously published work and current ROI analyses, processing in this region had appeared uninfluenced by univariate emotional information, though highly influenced by spatial information. The application of multivariate analysis techniques here appeared to provide additional sensitivity to identity-related changes in activity patterns between voxels in these regions that were not apparent amongst the gross changes in net activation of the region driven by location. This highlights the utility and complimentary nature of univariate and multivariate statistical techniques when analysing neuroimaging data.

It is important to note, however, that there is apparent increased specialization as information moves to more tertiary auditory processing areas (Alain et al. 2001; Ahveninen et al. 2006, 2013). Both univariate and multivariate analyses found that spatial information was being processed independently of emotion in later areas of the dorsal visual stream during the localization of naturalistic sounds, including areas of IPL and the post-central gyrus. This result followed predictions, as these regions are consistently implicated in multimodal integration of sensory information (Thiebaut de Schotten et al. 2005; Sack 2009) and spatial attention (Simon et al. 2002; Yantis et al. 2002).

While the current study increases our understanding of the representation of emotional information in auditory processing areas, as well as the complementary roles of univariate and multivariate statistical techniques on neuroimaging analyses, it does not address the mechanisms by which emotional information influences orienting behaviours associated with emotional stimuli (Bertels et al. 2010; Bradley et al. 2012). This work, similar to studies conducted in the visual domain (Kryklywy and Mitchell 2014), suggest that emotional information does not directly impact processing in tertiary spatial-processing auditory regions, yet rapid and seemingly automatic adjustments to orienting behaviours towards emotional sounds are often reported (Bertels et al. 2010; Bradley et al. 2012; Paulmann et al. 2012; Deuter et al. 2013). Additional work is still required to more clearly delineate the mechanisms by which this behaviour effect may occur.
